# An Apparatus for the Intratracheal Administration of Ether

**Published:** 1913-12

**Authors:** F. E. Shipway

**Affiliations:** Anæsthetist to Guy's Hospital.


### AN APPARATUS FOR THE INTRATRACHEAL ADMINISTRATION OF ETHER.

BY

F. E. SHIPWAY, M.A., M.D. Cantab.,

*Anæsthetist to Guy's Hospital.*

---

THIS is a modification of the apparatus which is pictured in the *British Journal of Surgery*, July, 1913, p. 105. It has been altered in many ways with the view to cut down weight and cost and to ensure simplicity. It consists of two parts: (A) the motor and blower; (B) the ether apparatus.

A.—The air current is supplied by an electric motor of $\frac{1}{8}$ h.p., driving a rotary blower, and is made to pass from the blower through an air filter, and then through a two-necked Wolff's bottle half-filled with water. The air filter serves the double purpose of picking up dust, etc., and of removing oil and grease. It consists of a rounded metal chamber, 5 inches high, $1\frac{1}{2}$ inches in diameter, with an entry tube at the bottom for connecting up to the blower, and an exit tube at the top issuing from a screw-on lid. Inside are three metal perforated discs fixed at equal distances upon a central rod, the lower end of which rests upon the bottom of the chamber; the upper end projects above the topmost disc and forms a handle for removal. Several layers of gauze are placed upon the discs, which fit accurately the interior of the chamber, so that all the air passes through them. This part of the apparatus is efficient, but it is my hope to combine it and the wash-bottle in some simple arrangement.

A resistance and speed-regulator controls the velocity of the air-current. A motor for a continuous current of 105 volts, driven from the main has been selected, since by placing a lamp in the circuit any continuous current up to 250 volts can be used. If the electric current breaks down, or is not suitable, or not available, a foot-bellows in conjunction with the wash-bottle must be used.

B.—The ether apparatus consists of an ether chamber, a warming chamber, and a mercury safety-valve, all connected together by metal tubing of $\frac{3}{8}$-in. bore.

The ether vessel is round and made of metal, with fixed lid, in the centre line of which are placed entry and exit tubes as far apart as possible. It has a diameter of 13 cm. and can take 30 ounces of ether when filled up to a mark on a glass gauge. There are also a funnel for filling and a tap for emptying the chamber. Metal has been chosen in preference to glass, as it is lighter, is not easily damaged, and can be made to any pattern or size. The chamber can be cleaned either by running hot water through it or by boiling after the glass gauge has been removed. The air-current passes over the surface of the ether.

The warming chamber is of the same size as the ether, and is also made of metal, with a loosely-fitting lid and an emptying tap. Inside is a coil of metal tubing. The exit tube is 3 inches in length, and bears a shielded thermometer and a tap for reducing the air-volume several times a minute.

Between these two chambers stands a glass mercury safety-valve, with a short arm projecting from the upper part at right-angles, and carrying a cup to prevent spilling of the mercury when the valve blows off. A manometer is an unnecessary encumbrance, as the safety-valve indicates the respiratory movements, and it is not essential to know the pressure which is being used at any given moment. It is only necessary to know that a certain safe pressure, say 20 mm. or 25 mm. Hg. cannot be exceeded.

A tap for connection with an oxygen cylinder is provided in the circuit before the ether chamber. This can also be used to reduce the air pressure from the blower, as it is my experience that in some cases the blower delivers either too small or too large an air-stream. Another way out of this difficulty is to use the motor at the "just-too-slow" speed, and supplement the current of air by a stream of oxygen. In all cases there is an optimum ventilation. Gas and oxygen can also be administered through this oxygen tap. I am at present working on this method.

The ether chamber is connected to the main tubing by the tap used by Elsberg, which has an entry and an exit tube, and consists of a central cog-wheel bearing a handle and gearing with two lateral cog-wheels. Each of these wheels bears a tap. By turning the handle any desired proportion of the air-current can be made to pass over the ether, or the air can be made altogether to miss the ether. A pointer on the handle shows the proportion of air and ether delivered to the patient, which is warmed up by its passage through the heating chamber.

The delivery tube from the latter is provided with 3 ft. of india-rubber tubing, the distal end being slipped over the catheter. Hot water is placed in the warming chamber, and the motor started before induction of anæsthesia. The tubes then are warmed up by the time the catheter has been passed, and generally speaking the thermometer stands at 120°–125°F. This temperature remains fairly constant for ten minutes, when fresh hot water must be added. An electric heating device may be used, but it has been thought desirable to make the outfit as simple as possible, and not too costly.

The ether apparatus is carried in a box of 3-ply wood, and has been made for me by the Surgical Manufacturing Company, 85 Mortimer Street, W. The electric direct laryngoscope and the linen-woven catheters have been made in accordance with my ideas by Messrs. Mayer & Meltzer, Great Portland Street, W.

